# Hierarchical Acoustic Encoding Distress in Pigs: Disentangling Individual, Developmental, and Emotional Effects with Subject-Wise Validation

**DOI:** 10.3390/ani16081148

**Published:** 2026-04-09

**Authors:** Irenilza de Alencar Nääs, Danilo Florentino Pereira, Alexandra Ferreira da Silva Cordeiro, Nilsa Duarte da Silva Lima

**Affiliations:** 1 Graduate Program in Production Engineering, Paulista University, Rua Dr. Bacelar 1212, São Paulo 04026-002, SP, Brazil; alexandracordeiro6@gmail.com; 2 Department of Management, Development, and Technology, School of Sciences and Engineering, São Paulo State University, Tupã 17602-496, SP, Brazil; danilo.florentino@unesp.br; 3 Department of Animal Science, Federal University of Roraima, BR 174, Km 12, Boa Vista 69310-000, RR, Brazil; nilsa.silva.lima@gmail.com

**Keywords:** pig welfare monitoring, bioacoustics, subject-wise validation, hierarchical variance partitioning, precision livestock farming

## Abstract

Automated pig-welfare monitoring needs scalable, non-invasive signals that work across ages and individuals. A key methodological contribution of this study is the use of subject-wise validation, which ensures generalization to unseen animals and prevents inflated accuracy caused by growth-related and individual ‘voice’ differences. Vocalizations can help, but growth and individual “voice” differences can confound distress patterns and overstate accuracy without subject-wise validation. In our study, we explicitly accounted for individual variability by including animal identity as a random effect in mixed models and by using grouped cross-validation, where models were tested only on pigs not seen during training. This approach ensures that the reported accuracy reflects generalization across different individuals rather than memorization of specific vocal signatures. We analyzed 2221 vocal samples from 40 pigs (20 males, 20 females) recorded across four growth phases (farrowing, nursery, growing, finishing) under six conditions (pain, hunger, thirst, cold stress, heat stress, normal). Acoustic features extracted in Praat included energy, duration, intensity, pitch, and formants (F1–F4). Using blockwise variance decomposition, we quantified contributions of distress exposure, growth phase, and sex, and estimated the additional variance explained by animal identity. Distress exposure dominated intensity and spectral traits, particularly Formant 2, whereas the growth phase produced systematic shifts in duration and pitch. Animal identity added a modest but consistent increment in explained variance (~+0.02–0.03 R^2^ beyond sex, phase, and distress). For prediction, we used 5-fold cross-validation grouped by animal. A Random Forest achieved a modest balanced accuracy of 0.609 and macro-F1 of 0.597; pain was most separable (recall 0.825), while other states showed moderate recall, indicating overlap. These results support hierarchical acoustic encoding of distress and establish a benchmark for precision welfare monitoring. Furthermore, they highlight that resolving complex physiological overlaps, such as heat stress and resource competition, requires a shift from unimodal acoustic models to multimodal Precision Livestock Farming (PLF) systems that integrate bioacoustics with continuous environmental and behavioral data streams.

## Simple Summary

Pig vocalizations can provide a non-invasive way to monitor welfare on farms, but the signals are influenced by more than “stress” alone. In this study, we analyzed over 2000 vocal recordings from 40 pigs across four growth stages and six conditions (normal, pain, hunger, thirst, cold stress, and heat stress). We found that some acoustic features, such as loudness-related measures and specific frequency bands, changed with distress. In contrast, other changes were strongly linked to growth stage (for example, differences in pitch and timing as pigs mature). In addition, each pig showed a consistent individual vocal pattern, meaning that models can look better than they really are if the same animals appear in both training and testing. Using a stricter evaluation that tested the model only on pigs it had not seen before, the system detected pain most reliably. Overall, pig vocalizations are useful for welfare monitoring, but robust systems must account for age-related shifts and individual differences.

## 1. Introduction

In commercial pig production, stress is frequently shaped by the housing environment and by routine management strategies rather than by a single isolated challenge. Environmental stressors such as thermal load, humidity, ventilation, stocking density, noise, and flooring interact with social dynamics and resource access, altering pigs’ behavioral options and physiological demands; consequently, animals may experience multiple concurrent stressors whose effects are difficult to disentangle using single indicators [1,2]. Management procedures, including regrouping/mixing, handling and restraint, transport, and other husbandry interventions, can elicit acute stress responses and cumulatively affect welfare, particularly when they are frequent or poorly adapted to the animals’ developmental stage and prior experience [3]. Because these environmental and managerial factors often co-occur and vary over time, stress expression in pigs should be conceptualized as context-dependent and multidimensional, motivating non-invasive monitoring approaches (e.g., bioacoustics) that can capture continuous changes under real housing and management conditions [1,2].

Animal stress is multidimensional (physiological, behavioral, contextual), and single markers or simple scores often provide only a crude assessment of a highly complex state [1,2,3]. Stress assessment in farm animals is further complicated by the fact that animals typically experience multiple concurrent stressors (social, environmental, and handling). In contrast, many experimental models impose only a single, controlled stressor, limiting ecological validity [1,3]. Classification of stress “severity” in routine animal research is also problematic: widely used score sheets reduce rich, multidimensional information to a few ordinal categories (mild/moderate/severe), and even trained personnel show substantial subjectivity and disagreement in recognizing and grading stress. This undermines standardization, ethical decision-making, and the comparability of studies [4,5].

In pigs specifically, traditional approaches that combine physiological sampling (e.g., cortisol) and observational ethograms are invasive, context-dependent, and confounded by sampling-induced stress and inter-individual variation, making reliable classification difficult in commercial settings [6,7]. This motivates the use of more continuous, non-invasive indicators (e.g., facial or acoustic features) but also raises modeling challenges because the underlying state is continuous and heterogeneous.

Conventional welfare and behavioral diagnostics typically classify problems or states into discrete categories (e.g., “separation anxiety”, “territorial aggression”, “low vs. high stress”), which is attractive for decision making but scientifically reductive. In companion animal behavioral medicine, categorical systems struggle because behavior is complex, multifactorial, and resistant to discrete labels; individuals with the same diagnosis can differ markedly in etiology, expression, and risk factors [8]. Applying a category risks losing the “uniqueness of the individual” and obscuring important variation.

Categorical stress classes also fail to respect the temporal dimension of stress (acute vs. chronic) and the fact that different metrics integrate different time scales, so assigning a single class ignores within-individual dynamics and measurement-specific windows [2]. In pig stress recognition, recent deep learning approaches still reduce complex emotional and physiological variation to binary labels (low/high stress in facial images), which is useful for automation, but cannot disentangle individual traits, developmental stages, or emotion types [6,7].

Previous studies often reported high accuracy in stress classification but did not account for the confounding effects of individual vocal signatures. Our study advances this field by foregrounding subject-wise validation as a methodological safeguard, ensuring that predictive models generalize to new animals rather than memorizing individual voices.

Hierarchical (multi-level) models are widely recommended to address these limitations. In clinical and behavioral research, hierarchical (including bifactor) models separate specific symptom variance from general distress, distinguishing general distress from domain-specific factors (e.g., fear) and improving interpretability and construct validity [9]. This framework readily applies to animal emotion, where a general distress factor can coexist with subordinate dimensions (such as arousal, context, or modality) alongside individual-level random effects. In affective computing and acoustic emotion recognition, hierarchical approaches capture structure across multiple levels, from frames to utterances and from low-level acoustic cues to higher-level affective representations. Hierarchical RNNs and attention-based architectures capture the temporal organization of signals and often outperform flat models in terms of robustness and accuracy [10,11,12]. Similarly, hierarchy-aware acoustic models integrate coarse- and fine-grained labels via multi-task learning, leveraging category structure to improve generalization [13].

Recent work in auditory affective neuroscience likewise decomposes complex audio into multi–level acoustic–semantic features and parallel components (e.g., voice vs. background) to model brain and behavioral emotion responses, revealing that different levels of representation contribute differentially to emotional encoding [14]. This provides a strong conceptual precedent for modeling pig vocalizations using hierarchical representations that span acoustic detail, individual identity, development, and emotional context.

In commercial pig production, welfare problems typically arise from housing or management failures that require rapid intervention rather than retrospective assessment. Early detection of heat stress and ventilation failure is a priority, as thermal load can escalate quickly under poor air exchange or equipment malfunction; acoustic alerts could support timely adjustments to ventilation, cooling, stocking density, or handling schedules [1,3]. Similarly, identifying water access failures, such as blocked drinkers or insufficient flow, could prompt targeted inspection and reduce dehydration risk that routine checks may overlook [1,2]. Detection of prolonged hunger or feeding competition addresses challenges related to feeder access, rationing, and social dominance, enabling management responses such as feeder adjustment or regrouping [1,3]. Finally, recognizing handling- or pain-related events could improve immediate mitigation and ethical oversight, particularly given the subjectivity of categorical severity scoring in practice [3,4,5]. These applied targets motivate a modeling strategy that separates distress from developmental stage, sex, and individual identity, while testing generalization to unseen pigs. Effective farm-level systems must remain robust to heterogeneous animals and shift growth baselines while still producing interpretable alerts tied to specific management risks.

In the context of Precision Livestock Farming (PLF), automated welfare monitoring must move beyond isolated sensor alerts. While bioacoustics provides a continuous, non-invasive stream of data, single-modality approaches often struggle to differentiate overlapping physiological states, such as the behavioral and acoustic similarities between heat stress and resource deprivation. Therefore, establishing a robust, hierarchy-aware acoustic baseline is a crucial first step. These acoustic models are intended to be integrated into broader computational pipelines that fuse vocal data with continuous environmental metrics and computer vision to provide reliable, multimodal decision support in commercial settings.

We aimed to partition the variance of pig vocal acoustic traits into emotional (distress), developmental (growth phase), sex, and individual components, and to benchmark multi-class distress classification using grouped cross-validation by animal to assess generalization to new individuals.

## 2. Materials and Methods

### 2.1. Data Source and Experimental Design

Vocalizations were obtained from 40 pigs (20 males and 20 females), individually identified (IDs 1–40) throughout the study. The experiment was conducted at a commercial pig farm in São Paulo State, Brazil (22°37′59″ S; 47°03′20″ W). Animals were housed in a solar house-oriented East–West (side height 3.5 m) with a 1 m lateral wall; side openings were partially open and controlled by polypropylene curtains, and the roof was covered with clay tiles. The farm managed the complete production cycle (gestation, farrowing, nursery, growing, and finishing).

The animals were hybrid pigs (a crossbreed of Landrace and Large White), selected for rapid growth and meat quality. This choice reflects the usual practice in commercial feedlot farms in Brazil. Although we did not compare specific breeds, we included growth phase and sex as fixed effects in the statistical models and individual identity as a random effect to control for physiological and behavioral differences potentially related to genetic background.

Recordings were performed across four growth phases: farrowing (1–4 weeks old), nursery (5–8 weeks old), growing (9–16 weeks old), and finishing (17–18 weeks old). In the husbandry description, pigs remained with sows during farrowing until weaning (≈21 days), were transferred to the nursery and remained there until approximately 60 days of age, and were subsequently relocated to the growing/finishing areas until reaching ~100 kg body weight. Each animal was evaluated under six conditions: heat stress, cold stress, hunger, thirst, pain, and normal (baseline). Cold stress was induced by turning off the heating systems; pigs were exposed to 25 °C for 30 min during farrowing and to 22 °C for 1 h during nursery. Heat stress was induced by turning off the axial fans and closing the side curtains to reduce ventilation, resulting in internal temperatures of approximately 27.5 °C in the growing house and 30 °C in the finishing house for 1 h. Hunger was induced by restricting nursing for 30 min during farrowing and by restricting feed access for 1 h during the nursery, growing, and finishing stages. Thirst was induced by restricting nursing for 30 min during farrowing and by restricting water access for 1 h across growth phases. Pain was induced by a mid-torso squeeze (e.g., firm restraint for vaccination), which the handler described as firmer than simple restraint. The normal condition represented standard rearing with thermal comfort and feed and water ad libitum, without pain induction.

The study used a repeated-measures design in which the vocalization of each animal was recorded for each distress condition during each growth phase; after each recording, observations were indexed by pig ID, growth phase, and distress exposure. For each test, sound was recorded for 60 s, and each 60 s signal was divided into three 20 s samples from which acoustic attributes were extracted. To reduce carryover effects, pigs were rested for at least 2 h between successive tests. This structure implies hierarchical nesting of measurements (Animal → Growth phase → Distress condition → Recording [60 s] → Sample [3 × 20 s]).

### 2.2. Sound Recording and Preprocessing

Vocalizations were recorded in the experimental pens using a unidirectional microphone (Yoga Electronics Co., HT-320a, Neihu District, Taipei, Taiwan) positioned approximately 20 cm from the animal during the test. The microphone was connected to a digital recorder (Marantz Japan Inc., PMD660/U3B Compact Flash recorder, Nisshin-cho, Kanagawa, Japan), and the acoustic signals were digitized at 44,100 Hz. For each test, the sound emitted by each pig was recorded continuously for 60 s. The recorded audio files were edited and analyzed using Praat^®^ version 6.1.09 [15]. Each 60 s recording was subdivided into three non-overlapping 20 s samples; from each sample, acoustic attributes were extracted for subsequent analysis. The extracted attributes comprised time-domain and spectral descriptors consistent with the source protocol, including (but not limited to) signal energy, call duration, maximum and minimum amplitude, intensity level, fundamental frequency (pitch), and formant frequencies (F1–F4).

The resulting feature table (File S1) was inspected for completeness and reasonableness of units across attributes (e.g., duration in seconds, intensity in dB, pitch/formants in Hz). Categorical variables (Animal identifier, sex, growth phase, and distress exposure) were encoded as factors for inferential models and as appropriately coded predictors for machine learning. Records with missing pitch values (expected in unvoiced segments or when pitch tracking fails) were retained for variance-partitioning analyses of other traits and handled explicitly in predictive models using a missing-data strategy defined a priori (complete-case for pitch-dependent models and/or imputation was justified). Continuous predictors were examined for extreme values; where needed, robust procedures were applied (e.g., winsorization at conservative tails or log-transformation of positively skewed variables such as duration and energy) to reduce undue leverage while preserving the biological signal. Finally, continuous predictors were standardized (z-scores) for models sensitive to scale, while tree-based models were trained on the original scale, as scaling does not affect split-based learning.

### 2.3. Missing Data Handling and Sensitivity Analyses

Pitch values were missing for a non-trivial proportion of samples, and the missingness varied across growth phase and distress condition. To avoid information leakage, all imputations were performed within each cross-validation fold: numeric features (including pitch and formants) were imputed using the median computed on the training fold only, and categorical variables were imputed using the most frequent category in the training fold. This strategy preserves the subject-wise generalization setting because the test fold contains only unseen animals, and they do not inform the imputation parameters.

Given the potential for structured missingness to bias inference (e.g., confounding phase × condition effects) and to affect prediction (models may exploit missingness patterns), we performed two sensitivity analyses: (i) a complete-case analysis restricted to samples with observed pitch, repeated under Animal-grouped cross-validation; and (ii) a “pitch-excluded” model in which pitch was removed from the predictor set, thereby eliminating any influence of pitch missingness on classification. The main results were considered robust if qualitative conclusions (pain separability, key confusions among non-pain stressors, phase modulation) persisted across sensitivity settings.

### 2.4. Statistical Analysis—Variance Partitioning

Because each pig contributed repeated vocal samples across multiple growth phases and distress exposures, acoustic variability may arise from (1) experimental condition (distress exposure), (2) developmental stage (growth phase), (3) sex, and (4) stable individual differences (“voice identity”). Moreover, each test comprised a 60 s recording segmented into three 20 s samples, creating an additional within-test clustering level (Recording → Sample). To disentangle these sources, we used a hierarchical variance-partitioning framework based on linear mixed-effects models, complemented by blockwise (factor-level) variance decomposition.

For each acoustic trait y (e.g., intensity, duration, pitch, formant frequencies), we fitted mixed-effects models with fixed effects for Sex, Growth phase, and Distress exposure, and random intercepts to account for repeated measurements within Animal. The core model is shown in Equation (1).

(1) $$y_{ijkm}=\beta_{0}+\beta_{1}{\mathrm{Sex}}_{i}+\beta_{2}{\mathrm{GrowthPhase}}_{j}+\beta_{3}{\mathrm{Distress}}_{k}+u_{i}+\varepsilon_{ijkm}$$

where $u_{i}∼N(0,\sigma_{Animal}^{2})$ captures stable between-animal differences and $\varepsilon_{ijkm}∼N(0,\sigma^{2})$ is the residual error. When supported by the data structure (i.e., when “Recording number” was available and represented the 60 s test), an additional random effect for recording was included to reflect the segmentation of each 60 s test into three samples (Sample nested within Recording, nested within Animal), as presented in Equation (2).

(2) (1∣Animal)+(1∣Animal:RecordingNumber)

This specification respects the repeated-measures design (Animal → Growth phase → Distress exposure → Recording → Sample). The Animal (Recording Number) represents the nesting of the three 20 s samples within a single 60 s recording session to avoid pseudo-replication

Intraclass correlation coefficients (ICCs) were used to quantify the proportion of residual variance attributable to stable between-animal differences (animal identity) after accounting for fixed effects, where $\sigma_{\mathrm{Animal}}^{2}$ is the between-animal variance component and $\sigma_{e}^{2}$ is the residual (within-animal) variance. To evaluate the generalization of unseen animals (deployment-relevant performance), we used grouped cross-validation with Animal as the grouping variable. Specifically, folds were constructed such that all samples from a given pig were assigned exclusively to either the training set or the test set within a fold (i.e., no animal appeared in both sets). This design provides a conservative estimate of performance in real-world use, where the model will be applied to pigs not represented in the training data.

The contribution of individual identity was summarized using (i) the Animal-level variance component *σ*^2^*_Animal_* and (ii) the intraclass correlation coefficient (ICC), computed as described in Equation (3).

(3) $${\mathrm{ICC}}_{Animal}=\sigma_{Animal}^{2}/(\sigma_{Animal}^{2}+\sigma^{2})$$

When a recording-level random effect was included, Equation (4) was applied.

(4) $${\mathrm{ICC}}_{Animal}=\sigma_{Animal}^{2}/(\sigma_{Animal}^{2}+\sigma_{Recording}^{2}+\sigma^{2})$$

Larger ICC values indicate stronger, trait-specific individual signatures that extend beyond experimental conditions and developmental stages.

Partitioning variance attributable to fixed factors (distress, growth, sex). To express the relative importance of distress exposure and growth phase in an interpretable manner, we reported marginal and conditional coefficients of determination for mixed models (Nakagawa-type R^2^: marginal R^2^ (R^2^_m_)) reflects variance explained by fixed effects (Sex, Growth phase, Distress exposure), whereas conditional R^2^ (R^2^_c_) reflects variance explained by both fixed and random effects (including Animal identity). The incremental contribution of Animal identity can then be expressed as (R^2^_c_ − R^2^_m_), interpreted as the additional explained variance attributable to stable between-animal differences after accounting for Sex, Growth phase, and Distress exposure.

In addition, blockwise variance decomposition for the fixed effects was applied, using reduced-model comparisons. Specifically, for each trait, we fitted the full fixed-effects model (Sex, Growth phase, Distress exposure). Then we refitted reduced models by removing one block at a time (e.g., removing Distress exposure while retaining Sex and Growth phase). The importance of each block was summarized as the reduction in explained variance relative to the full model (ΔR^2^) or semi-partial (R^2^), depending on the implementation, thereby quantifying the unique contribution of distress and developmental stage to each acoustic trait.

To assess whether distress signatures were development-dependent, we evaluated biologically plausible interaction terms (Distress exposure × Growth phase). We retained them when they improved model fit, as assessed by likelihood ratio tests and information criteria (AIC), while preserving parsimony. Residual diagnostics were examined for normality and homoscedasticity; traits with marked skewness (commonly duration and energy) were log-transformed before modeling when appropriate. For traits with missing values (notably pitch, due to unvoiced segments or tracking failure), models were fitted using all available observations for each trait (trait-wise analysis), and missingness patterns were summarized descriptively to support transparent interpretation.

Because variance-partitioning was performed across multiple acoustic traits, we controlled the false discovery rate for families of hypothesis tests using the Benjamini–Hochberg procedure when interpreting inferential *p*-values across traits. For all traits, we report effect estimates, variance components, (R^2^_m_)/(R^2^_c_), ICC, and confidence intervals where applicable, prioritizing effect sizes and uncertainty over reliance on statistical significance alone. To summarize explained variance, we report the marginal coefficient of determination ($R_{m}^{2}$), defined as the proportion of variance explained by fixed effects only, and a conditional R^2^ quantity ($R_{c}^{2}$), intended to reflect the proportion of variance explained by both fixed effects and animal identity.

### 2.5. Predictive Modeling Under Subject-Wise Generalization

A supervised multi-class classification task was defined to predict distress exposure (pain, hunger, thirst, cold, heat stress, normal) from acoustic features. The response variable was the categorical “Distress exposure” label recorded for each sample, and all models were trained to output either class labels or class probabilities for the six conditions.

Predictors comprised the extracted acoustic descriptors (signal energy, duration, maximum and minimum amplitude, intensity, pitch, and formants F1–F4, including derived formant metrics when available). Sex and growth phase were included as additional predictors in the main analysis to reflect biologically meaningful developmental and sex-related modulation of vocal output. A sensitivity analysis excluding sex and growth phase was planned to assess the extent to which distress discrimination can be achieved from acoustics alone (i.e., without relying on metadata that may not be available in field deployments).

Before modeling, continuous predictors were screened for implausible values and extreme outliers; when needed, robust transformations were applied to reduce leverage while preserving rank structure (e.g., log transformations for positively skewed variables such as duration and energy). Continuous predictors were standardized (z-scored) for models sensitive to feature scaling (e.g., linear classifiers), whereas tree-based models were trained on the original scale. Missing values were handled explicitly. In particular, pitch can be missing in unvoiced segments or when tracking fails; therefore, predictive models were fit using (i) a complete-case approach for analyses requiring pitch and (ii) a missing-data strategy that preserves sample size when feasible (e.g., median imputation within training folds only, or use of models tolerant to missingness if implemented). All imputation and scaling operations were performed within each training fold to prevent information leakage.

To evaluate generalization to unseen animals (deployment-relevant performance), we used grouped cross-validation with Animal as the grouping variable. Specifically, folds were constructed such that all samples from a given pig were assigned exclusively to either the training set or the test set within a fold (i.e., no animal appeared in both sets). Performance was estimated using 5-fold grouped cross-validation; fold assignments were stratified where possible to reduce class imbalance across folds while maintaining the strict grouping constraint. This design provides a conservative estimate of performance in real-world use, where the model will be applied to pigs not represented in the training data.

We used logistic regression for interpretability and Random Forest for non-linear performance, prioritizing clarity and robustness over more complex alternatives, in contrast to studies that frequently explore advanced architectures and optimization strategies for complex classification problems [16]. The baseline model was a multinomial linear classifier (e.g., multinomial logistic regression with regularization), which provides a transparent benchmark. The primary non-linear model was a tree-ensemble classifier (Random Forest), selected for its ability to capture non-linear relationships and interactions among acoustic descriptors without strong parametric assumptions. Hyperparameters were tuned using a cross-validated search conducted strictly within the training portion of each grouped fold (nested tuning) or, when computational constraints required, using a prespecified hyperparameter grid optimized via grouped cross-validation on the training set only. Class imbalance was addressed either by class weighting (preferred for linear models) and/or by balanced subsampling strategies within tree ensembles; the chosen strategy was held constant across folds.)

Performance metrics and reporting. Because distress classes may be imbalanced, overall accuracy alone can be misleading. Therefore, performance was quantified using balanced accuracy (the mean of class-wise recalls) and the macro-average F1-score (giving equal weight to each class), along with class-wise precision, recall, and F1-score. Confusion matrices were computed for each fold and aggregated (mean-normalized) to identify systematic confusions across distress exposures. Where probabilistic outputs were available, one-vs-rest AUROC and calibration summaries (e.g., reliability curves or Brier score) were optionally reported, focusing on whether model confidence tracks empirical correctness.

To support biological interpretation and model transparency, feature contributions were assessed using permutation importance computed within the grouped cross-validation framework (i.e., estimated from held-out-fold predictions). For the primary model, the stability of importance rankings across folds was summarized to identify robust predictors. If included, complementary methods, such as SHAP values, were computed on the training-fitted models and summarized at the fold level to describe directionality (e.g., whether higher intensity or higher pitch increased the probability of specific distress states).

We conducted three additional analyses to validate the model. First, we refit the model using acoustics-only predictors (excluding sex and growth phase) to determine how much it relied on metadata. Second, we compared complete-case handling with within-fold imputation to evaluate sensitivity to missing pitch data. Third, we calculated metrics for each growth phase separately to see if model generalization varied across developmental stages.

Software implementation details. All modeling steps (encoding, scaling, imputation, hyperparameter search, training, and evaluation) were implemented using a pipeline-based approach to ensure that preprocessing was fitted exclusively on training data within each cross-validation fold and then applied to the corresponding test data, thereby preventing leakage and yielding unbiased performance estimates under subject-wise validation.

### 2.6. Confusability/Overlap Analysis

Previous work on distress recognition in pigs has highlighted partial overlap among stress-related classes in acoustic feature space, which can lead to systematic misclassification even when overall accuracy appears acceptable. Because our objective was not only to predict distress exposure but also to understand failure modes under realistic deployment conditions (unseen animals), we conducted a dedicated confusability/overlap analysis using out-of-sample predictions generated via subject-wise cross-validation.

For the best-performing model (and, where relevant, the baseline model), we computed a normalized confusion matrix using held-out predictions from grouped cross-validation. Each row (true class) was normalized to sum to 1, yielding per-class error distributions. Confusability between two distress states *a* and *b* was summarized using (i) directional confusion C(*a*→*b*) (probability of predicting *b* when the true class is a) and (ii) a symmetric confusability index (Equation (5)).


(5) $$S(a,b)=\frac{1}{2}[C(a\to b)+C(b\to a)]$$

This symmetric index enables ranking of the most mutually confusable class pairs and provides an interpretable “overlap map” that is robust to class imbalance.

To move beyond hard class assignments, we quantified probabilistic overlap using the vector of predicted class probabilities. *p*_*i*_ for each sample *i*. Two complementary measures were derived from held-out predictions:

(i) Prediction entropy (uncertainty) (Equation (6)).

(6) $$H_{i}=-\sum_{k=1}^{K}p_{\mathrm{ik}}\log(p_{\mathrm{ik}})$$

where K = 6 distress classes. Higher entropy indicates diffuse probability mass (greater ambiguity), consistent with overlapping acoustic signatures.

(ii) Margin (confidence gap) (Equation (7)).

(7) $$M_{i}=p_{i(1)}-p_{i(2)}$$

where $p_{i(1)}$ and $p_{i(2)}$ are the highest and second-highest predicted probabilities. Smaller margins indicate closer competition between the two classes and are expected to overlap.

For each distress exposure, we summarized the distributions of entropy and margin (median and IQR) and compared classes using nonparametric tests as appropriate. This allowed us to distinguish “true separability” (high recall, low entropy, large margins) from “apparent separability” driven by class imbalance or dominant predictors.

Because vocal production changes across growth phases, we evaluated whether confusability patterns were phase dependent. Specifically, we recalculated the normalized confusion matrix and the symmetric confusion index. S(a,b) and uncertainty metrics (entropy and margin) separately within each growth phase (farrowing, nursery, growing, finishing) using only the held-out samples from that phase. This stratification tests the hypothesis that overlaps among distress states are modulated by developmental stage and identifies phase-specific misclassification risks that are critical for on-farm implementation.

To provide an interpretable summary of multi-class overlap, we constructed a class-level similarity matrix S(a,b). Moreover, visualized it as (i) a heatmap and (ii) a network graph in which nodes represent distress classes and edge weights represent symmetric confusability. Hierarchical clustering of the similarity matrix was used to identify “overlap clusters” (groups of distress states with mutually elevated confusions). Cluster stability was assessed across cross-validation folds to ensure that a single fold did not drive identified overlap structures.

Two robustness analyses were performed to verify that confusability patterns reflected acoustic overlap rather than reliance on metadata. First, overlap analyses were repeated using an acoustics-only model, excluding sex and growth phase, to determine whether confusions persist when only sound descriptors are available. Second, overlap analyses were repeated under alternative missing-pitch handling strategies (complete-case vs. within-fold imputation) because pitch availability can influence separability among arousal-related states. Across analyses, we report consistent confusable pairs/clusters as evidence of genuine acoustic overlap, and we interpret phase-specific changes as developmental modulation of distress encoding.

The confusability/overlap analysis is reported alongside overall performance metrics to highlight where predictions are reliable (high recall, low entropy) versus ambiguous (low recall, high confusability, high entropy). These results motivate future improvements (e.g., call-type annotation, raw audio embeddings, or multimodal integration) to reduce overlap among non-pain distress states under real-world conditions.

### 2.7. Software and Reproducibility

The analytical dataset was assembled as a tabular file (File S1: database.xlsx) containing the acoustic descriptors and experimental factors (Animal, sex, growth phase, distress exposure, recording/sample identifiers). All subsequent statistical analyses, variance-partitioning models, and predictive modeling were implemented in a fully scripted workflow in Python v.3.11.2. Data manipulation used pandas 2.2.3, and NumPy 1.24.0; supporting statistics used SciPy 1.14.1; inferential regression and mixed-effects modelling used statsmodels 0.14.3; and machine-learning models, grouped cross-validation, and performance metrics were implemented in scikit-learn 1.4.2. Figures were generated using Matplotlib 3.7.5.

To ensure reproducibility and prevent information leakage, all preprocessing steps (categorical encoding, scaling, and imputation of missing values) were performed within a pipeline trained exclusively on the training portion of each cross-validation split, then applied to the corresponding held-out fold. Subject-wise generalization was enforced by constructing folds with Animal as the grouping variable, ensuring that no individual appeared in both the training and test sets within a fold. All stochastic procedures (e.g., model bootstrapping, feature subsampling, and fold shuffling where applicable) were controlled by fixed random seeds, which are reported in the analysis script.

Reproducibility artifacts include: (i) a single executable analysis script (or notebook) that regenerates all tables and figures from the File S1; (ii) a machine-readable configuration file specifying model hyperparameters and cross-validation settings; and (iii) exported intermediate outputs (e.g., fold-wise predictions and confusion matrices) to enable independent verification of reported performance and overlap analyses.

### 2.8. Model Explainability and Biological Interpretation of Predictors

To improve interpretability beyond aggregate performance metrics, we quantified feature contributions for the best-performing subject-wise classifier using permutation importance under grouped cross-validation. Briefly, within each Animal-grouped test fold, each predictor was randomly permuted (one at a time) in the held-out set while preserving the joint distribution of the remaining predictors, and the performance decrement was computed as the change in macro-averaged F1 score (Δmacro-F1). This procedure yields an out-of-fold importance estimate that is robust to within-individual correlation structure because all test samples come from animals not seen during training. Fold-specific importances were aggregated across the five grouped folds, and uncertainty was summarized using the fold-to-fold distribution (mean ± 95% confidence interval derived from the empirical percentiles across folds). Importances were computed for numeric acoustic features (signal energy, window-level duration, intensity, pitch, formants 1–4, and amplitude extrema) and categorical covariates (sex and growth phase, represented as one-hot encodings). The resulting rankings were used to guide a biologically constrained interpretation of distress separability, emphasizing plausible pathways, including respiratory effort and phonation control under thermal load, arousal-related changes in amplitude and spectral energy distribution, and growth-related shifts in resonant structure, as reflected in formant trajectories.

## 3. Results

### 3.1. Descriptive Statistics and Data Completeness

Table 1 summarizes the distribution of samples by growth phase, distress exposure, and sex, and reports pitch missingness by cell. Overall, the dataset comprised 2221 vocal samples from 40 pigs, with a balanced sex distribution (Female: 1104; Male: 1117). The number of samples per growth phase was Farrowing (*n* = 482), Nursery (*n* = 595), Growing (*n* = 595), and Finishing (*n* = 549). Across distress exposures, sample sizes were Pain (*n* = 475), Normal (*n* = 472), Hunger (*n* = 454), Thirst (*n* = 351), Cold (*n* = 246), and Heat stress (*n* = 223). Not all distress exposures were represented in every growth phase (e.g., no Heat stress samples were present in Farrowing or Nursery).

Pitch missingness was strongly structured rather than random. In a logistic regression of “pitch missing” (yes/no) on growth phase and distress exposure (reference categories: Farrowing and Normal), the odds of missing pitch increased markedly with development, particularly in the Finishing phase (OR = 28.26; 95% CI: 20.99–38.82), and also in the Growing phase (OR = 6.30; 95% CI: 4.23–8.70) and Nursery phase (OR = 2.61; 95% CI: 1.52–3.71). Distress exposures also contributed: heat stress was associated with higher odds of missing pitch relative to Normal (OR = 4.03; 95% CI: 2.45–7.21), as was hunger (OR = 2.76; 95% CI: 1.71–4.70) and the noxious handling/pain condition (OR = 1.72; 95% CI: 1.24–2.41). In contrast, thirst and cold stress were associated with lower odds of pitch missingness relative to Normal (Thirst OR = 0.34; 95% CI: 0.15–0.66; Cold OR = 0.30; 95% CI: 0.24–0.39). These results indicate that pitch missingness reflects systematic differences in signal properties across both development and condition (e.g., increased unvoiced segments/noise or reduced stable voicing), rather than merely measuring noise.

Sensitivity analyses confirmed that the main classification conclusions were not driven solely by pitch-related missingness. Under Animal-grouped cross-validation, the full model with within-fold imputation achieved a balanced accuracy of 0.610 and a macro-F1 of 0.595 (N = 2221). In the complete-case analysis (observed pitch only), performance was similar (balanced accuracy = 0.612; macro-F1 = 0.605; N = 1980). When pitch was excluded from the predictor set, performance decreased (balanced accuracy = 0.588; macro-F1 = 0.578; N = 2221), indicating that pitch contains useful information for distress recognition, but that the overall conclusions are not dependent on retaining pitch-missing samples via imputation.

The dataset comprised 2221 samples from 40 pigs with a balanced sex distribution (female: 1104; male: 1117), and similar sample sizes across growth phases (farrowing: 482; nursery: 595; growing: 595; finishing: 549). Across distress exposures, sample counts were highest for pain, normal, and hunger, and lowest for cold and heat stress; heat stress was not represented in the farrowing and nursery phases. Pitch completeness was high overall (missing in 10.9% of samples). However, it showed pronounced phase and condition-dependence: missingness was minimal in farrowing and nursery, increased in growing, and was highest in finishing. In terms of distress exposure, the missing pitch was greatest under heat stress and hunger, and lowest under cold and thirst. These patterns indicate that pitch-based comparisons require explicit handling of missing data and sensitivity analyses, particularly during late growth phases and under heat-stress and hunger conditions. In contrast, intensity, duration, and formant-based analyses can be conducted on the full dataset with minimal information loss.

### 3.2. Distress-Associated Acoustic Differences (Descriptive Statistics and Effect Sizes)

Table 2 summarizes the distribution of key acoustic traits across distress exposure levels. Descriptively, pain was characterized by markedly higher intensity and higher Formant 2 than the normal condition, whereas heat stress showed the highest mean pitch but the shortest mean duration. Cold stress was associated with the longest calls (duration) but comparatively low mean pitch and lower Formant 2. Pitch completeness varied by condition, with the greatest missingness under heat stress (34.5%), indicating that pitch-based contrasts involving heat stress should be interpreted with explicit consideration of missing data (Table 2).

**Figure 1:**
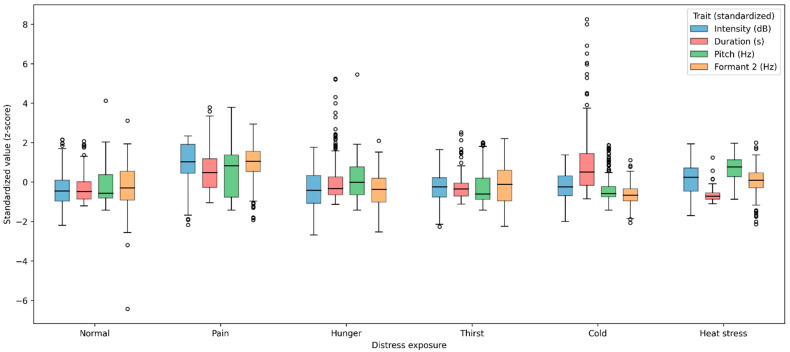
Distributions of standardized acoustic traits across distress exposures.

Figure 1 presents the trait distributions by distress exposure for intensity, duration, pitch, and Formant 2 (optionally stratified by growth phase). The plots highlight (i) a pronounced right-shift in intensity under pain relative to other conditions, (ii) a strong duration increase under cold stress, and a duration decrease under heat stress, (iii) a marked pitch elevation under heat stress, and (iv) higher Formant 2 values under pain, with partial distributional overlap among non-pain conditions.

Boxplots show z-score–standardized values of intensity, call duration, pitch, and Formant 2 for each distress condition (normal, pain, hunger, thirst, cold, heat stress). Boxes represent the interquartile range, central lines represent medians, whiskers extend to 1.5 × IQR, and points represent outliers. Pitch values were missing for a subset of samples (notably under heat stress and in the finishing phase), so pitch boxplots reflect only the available observations.

To quantify the magnitude of key contrasts without over-interpreting inferential significance, we computed Cohen’s d (descriptive effect sizes) for selected comparisons (positive values indicate higher means in the first condition). The largest effects were observed for pain relative to normal in intensity (d = 1.58), duration (d = 1.13), and Formant 2 (d = 1.34), and for cold relative to normal in duration (d = 1.26). Heat stress relative to normal showed a large increase in pitch (d = 1.03) coupled with shorter duration (d = −0.61). Collectively, these descriptive patterns suggest that pain and thermal challenges (cold/heat stress) yield particularly strong shifts in specific acoustic dimensions (intensity/formant structure for pain; duration for cold; pitch and shortened duration for heat stress), while the remaining states exhibit more moderate separation and greater overlap in their univariate distributions.

### 3.3. Growth Phase Effects and Developmental Modulation

**Figure 2:**
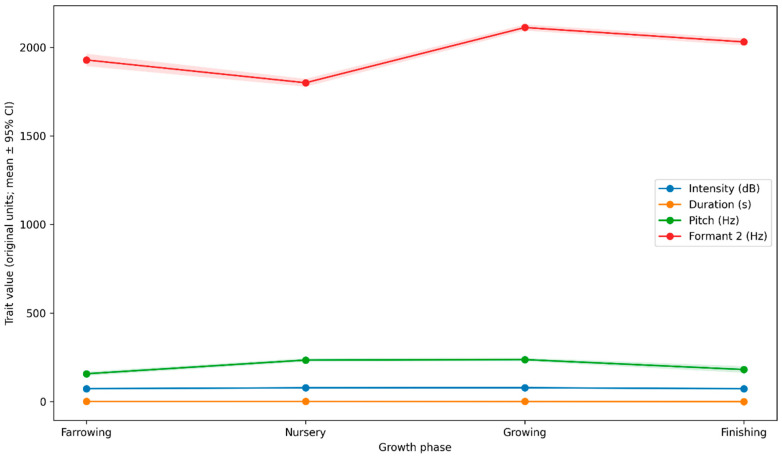
Growth-phase trajectories of key acoustic traits (mean ± 95% confidence interval). Mean values of intensity, call duration, pitch, and Formant 2 are shown across the four growth phases (farrowing, nursery, growing, finishing). Shaded bands represent 95% confidence intervals computed from the phase-specific standard errors.

Across growth phases, the acoustic profile of pig vocalizations showed marked developmental shifts (Figure 2). Mean call duration decreased progressively with age, from longer calls in farrowing and nursery to shorter calls in finishing, consistent with great stage-dependent changes in the temporal structure of vocal output. In contrast, mean pitch exhibited a non-monotonic trajectory: pitch increased from farrowing to nursery/growing and then decreased in finishing. However, uncertainty widened at the end due to higher pitch-missingness in late-phase samples. Formant 2 also varied systematically across development, with lower mean values in the nursery and higher values in the growing/finishing stage, indicating developmental modulation of spectral structure. Mean intensity exhibited comparatively smaller phase-to-phase shifts than duration and spectral features did; however, it remained measurably phase-dependent.

Developmental modulation was also evident in phase-stratified contrasts between distress exposures. For example, the pain-related elevation in intensity relative to normal was largest in early phases (approximately +11.8 dB in farrowing and +13.5 dB in nursery), remained substantial in growing (+10.4 dB), and attenuated in finishing (+5.0 dB), suggesting that the acoustic separation of pain vs. baseline becomes less pronounced later in development. Similarly, the pain-related increase in Formant 2 relative to normal was strongest in farrowing (+838 Hz) and nursery (+474 Hz). It diminished during growth (+67 Hz) and during finishing (+116 Hz), indicating that distress-related spectral shifts are phase-dependent. Conversely, heat-stress effects on pitch (heat-stress minus normal) were observed only during the heat-stress phases (growing and finishing). They were larger in finishing (+185 Hz) than in growing (+93 Hz), consistent with an amplification of heat-associated pitch elevation in late development. Taken together, Figure 2 indicates that the growth phase not only shifts the baseline acoustic phenotype (especially duration, pitch, and formant structure) but also modulates the apparent magnitude of distress signatures, supporting the need for phase-aware modeling and for evaluation designs that avoid confounding developmental and emotional effects.

### 3.4. Variance Partitioning and Individual Signatures (Primary Contribution)

**Figure 3:**
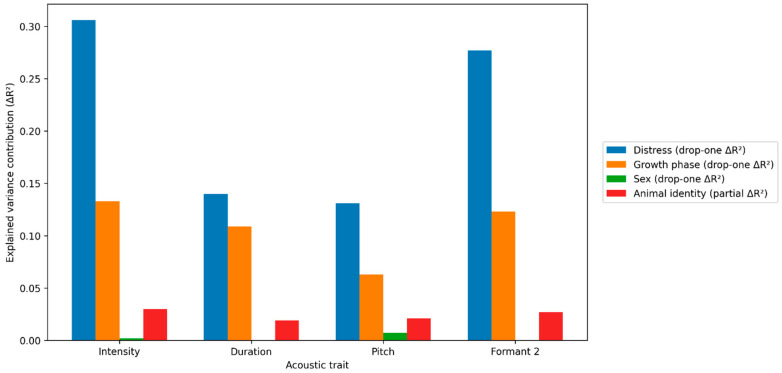
Relative contributions of distress exposure, growth phase, sex, and individual identity to explained variance across acoustic traits. Bars show drop-one ΔR^2^ contributions for fixed effects (distress exposure, growth phase, sex) and the partial ΔR^2^ attributable to animal identity after accounting for fixed factors.

Figure 3 summarizes the variance-partitioning results for the main acoustic traits. Across traits, distress exposure was the dominant contributor to explained variance, particularly for intensity and Formant 2 (drop-one ΔR^2^ = 0.306 and 0.277, respectively), while growth phase also contributed substantially (ΔR^2^ ≈ 0.063–0.133).

Table S1 (in the Supplementary Materials) reports the marginal intraclass correlation coefficient (ICC) for animal identity. $R^{2}$($R_{m}^{2}$) for fixed effects, and a conditional $R^{2}$ metric ($R_{c}^{2}$) capturing additional explained variance attributable to animal identity, along with drop-one Δ*R*^2^ contributions for distress exposure, growth phase, and sex.

Sex effects were comparatively small in magnitude across traits (ΔR^2^ ≤ 0.007), reaching statistical relevance only for intensity and pitch after false-discovery-rate adjustment. Individual identity contributed a consistent, non-negligible increase in explained variance after accounting for sex, growth phase, and distress (partial ΔR^2^ ≈ 0.019–0.030), indicating stable animal-specific signatures that persist across experimental conditions and developmental stages. Residual-based ICC estimates (computed on residuals after removing fixed effects) further supported detectable individual structure, with ICC values of 0.037 for intensity and 0.031 for Formant 2, and smaller ICC values for duration (0.012) and pitch (0.007), suggesting that “voice identity” is expressed more strongly in intensity and spectral structure than in pitch once fixed factors are controlled.

### 3.5. Subject-Wise Distress Classification Performance (Unseen Animals)

Table 3 and Table 4 report the subject-wise (Animal-grouped) cross-validation performance for three classifiers. Under this ‘unseen-animal’ evaluation, the Random Forest achieved the best overall generalization with a balanced accuracy of 0.609 and a macro-F1 of 0.597. These values indicate that, while the model captures relevant acoustic patterns, its performance remains limited, highlighting the challenge of distinguishing overlapping distress states. This outperformed the multinomial logistic regression baseline (macro-F1 = 0.498), underscoring the need to capture non-linear acoustic interactions and feature dependencies to achieve robust generalization across individuals.

At the class level (Random Forest), pain was the most separable distress state, with high precision (0.832) and recall (0.825), indicating detection of pain-associated vocal profiles under subject-wise generalization (Table 5). Cold and heat stress showed moderate recall (0.663 and 0.637, respectively). In contrast, hunger, thirst, and the normal baseline condition exhibited modest recall, equivalent to random recall (0.509, 0.556, and 0.464), consistent with structured overlap among non-pain states and with “normal” calls being partially confusable with mild distress conditions.

**Figure 4:**
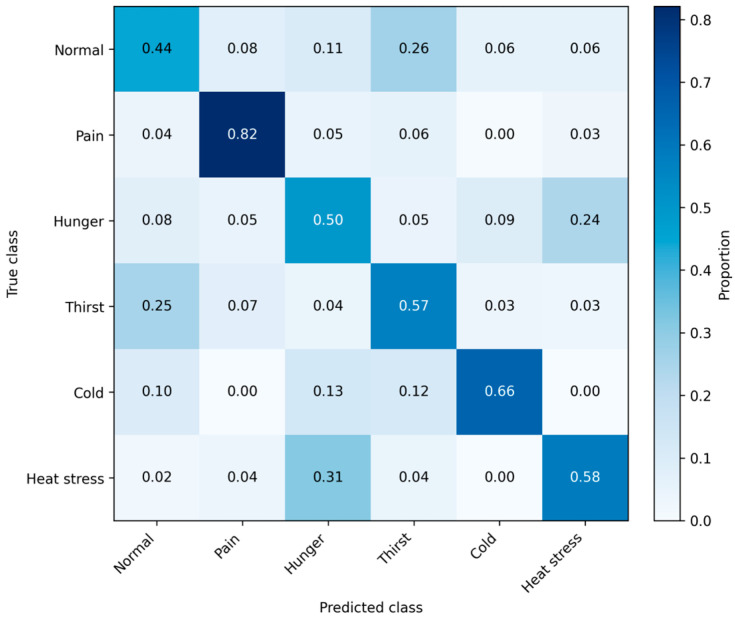
Subject-wise (unseen-animal) confusion matrix for distress classification. Row-normalized confusion matrix (rows sum to 1.00) obtained from pooled out-of-fold predictions using 5-fold cross-validation grouped by Animal for the best-performing model (Random Forest). Cell values represent the proportion of samples in each true distress exposure that are predicted to each class, thereby visualizing systematic confusion and overlap among distress states under strict generalization to unseen individuals.

Figure 4 presents the aggregated, row-normalized confusion matrix from the grouped (Animal-wise) cross-validation for the best-performing model, highlighting systematic confusion among non-pain distress states under unseen-animal generalization.

Figure 4 indicates that, under strict subject-wise generalization (unseen animals), the model reliably detects some distress states but also exhibits structured overlap with others. Pain is the clearest class. Most pain samples are correctly classified (high diagonal value for Pain), and misclassification is low across other classes. This supports the conclusion that pain-related calls occupy a distinct region of the feature space in this dataset, consistent with the large descriptive shifts previously observed for intensity and Formant 2.

In contrast, the non-pain conditions show systematic, non-random confusions that reveal genuine acoustic similarity (or label/context overlap) rather than noise. The most prominent pattern is bidirectional confusion between Hunger and Heat stress, indicating that these two states share acoustic characteristics captured by the current feature set (e.g., arousal-related changes affecting pitch/intensity), making them difficult to disentangle using engineered features alone. A second salient pattern is that Normal is frequently predicted as Thirst, suggesting that baseline vocalizations can resemble thirst-related calls (or that thirst induction produces comparatively subtle acoustic deviations), thereby reducing specificity for identifying “no distress”. Cold stress is moderately well-separated but still shows overlap with adjacent states, consistent with cold-induced changes being expressed strongly over time yet not uniquely diagnostic across contexts.

Three interpretive points for the manuscript are supported by the output in Figure 4: (i) pain is robustly identifiable across animals, (ii) thermal and resource-related stressors form an overlap cluster (especially hunger and heat stress), and (iii) the boundary between “normal” and milder distress (notably thirst) is porous, implying that future improvements should emphasize reducing these specific confusions, e.g., incorporating call-type labels, using raw-audio embeddings, adding contextual/environmental covariates (temperature, access to water/feed), or adopting hierarchical classification (pain vs. non-pain, then subtype within non-pain).

### 3.6. Overlap Structure and Phase-Stratified Confusions

We operationalized “confusability” as the symmetric off-diagonal agreement between two classes, $S(a,b)=\frac{1}{2}\{P(\hat{y}=b|y=a)+P(\hat{y}=a|y=b)\}$, computed from pooled out-of-fold predictions under Animal-grouped cross-validation. This yields an interpretable, undirected overlap map in which larger values indicate stronger bidirectional confusion.

**Figure 5:**
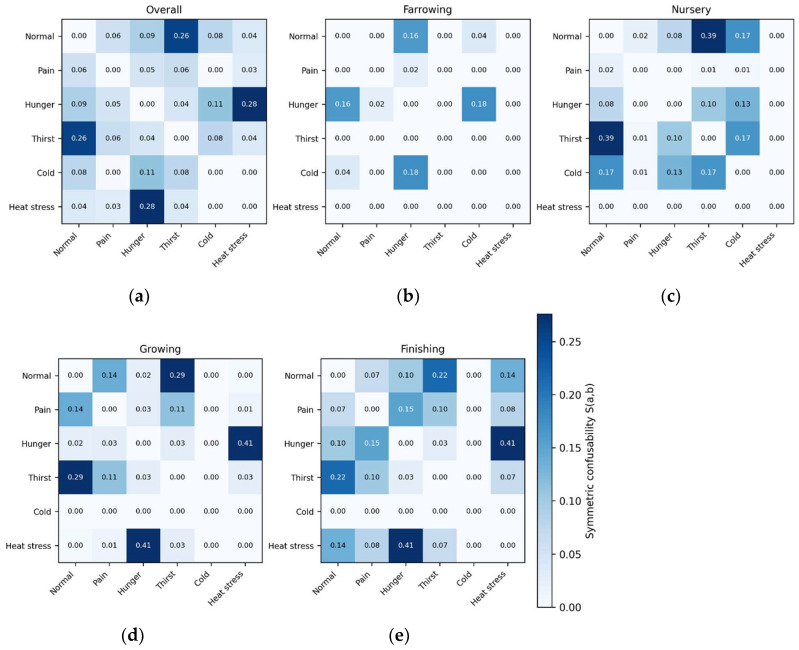
Confusability structure of distress classes under subject-wise generalization, overall, and stratified by growth phase: (**a**) Overall, (**b**) Farrowing, (**c**) Nursery, (**d**) Growing, and (**e**) Finishing.

Figure 5 presents a structured overlap topology rather than uniform error. Overall, the strongest confusability cluster was Hunger–Heat stress (S = 0.248), followed by Normal–Thirst (S = 0.242), with a secondary overlap between Hunger–Cold (S = 0.122). These pairings indicate that the model’s errors are concentrated in biologically plausible “arousal/physiological demand” neighborhoods (resource limitation and thermal load). In contrast, pain remains comparatively isolated from the principal overlap clusters.

Heatmaps in Figure 5a–e show symmetric confusability. S(a,b), computed from pooled out-of-fold predictions (Animal-grouped cross-validation) of the best-performing model. Values represent bidirectional overlap between class pairs; diagonal entries are omitted. Phase panels display confusability within each growth phase, highlighting developmental modulation of class overlap.

The growth phase substantially modulated the overlap map. In Farrowing, the dominant confusion cluster shifted toward Hunger–Cold (S = 0.193) and Normal–Hunger (S = 0.146), consistent with early-life calls exhibiting strong temporal and spectral similarity across baseline and mild distressors. In Nursery, Normal–Thirst showed the largest overlap (S = 0.356), accompanied by elevated Normal–Cold (S = 0.187) and Thirst–Cold (S = 0.172), suggesting that, in this phase, the boundary between baseline and non-pain challenges is particularly porous. In Growing, the Hunger–Heat stress overlap intensified markedly (S = 0.385), while Normal–Thirst remained prominent (S = 0.298); additional overlap emerged for Pain–Thirst (S = 0.121) and Normal–Pain (S = 0.116), indicating that mid-development increases both the strength and diversity of overlap pathways. In Finishing, Hunger–Heat stress remained a dominant cluster (S = 0.358), and Normal overlapped comparably with Thirst (S = 0.193) and Heat stress (S = 0.192), while Pain–Hunger (S = 0.119) and Pain–Thirst (S = 0.098) became more evident than in earlier phases, consistent with attenuated pain separability observed in the variance-partitioning patterns.

These phase-dependent overlap patterns aligned with the model’s uncertainty profile derived from out-of-fold class probabilities. Median predictive entropy increased across development (Farrowing: 0.745; Nursery: 1.227; Growing: 1.115; Finishing: 1.393), while the median probability margin (top–second class) decreased (Farrowing: 0.555; Nursery: 0.230; Growing: 0.206; Finishing: 0.166), indicating progressively less confident discrimination in later phases. By class, pain showed the lowest entropy and highest margin (entropy median: 0.527; margin median: 0.785), whereas thirst and heat stress exhibited higher entropy and lower margins (thirst margin median: 0.152; heat stress margin median: 0.162), consistent with their placement within the principal overlap clusters in Figure 5. Collectively, Figure 5 supports the interpretation that distress encoding is not only multidimensional but developmentally reweighted, with late-phase vocal phenotypes increasing overlap among non-pain distressors and reducing classification certainty.

### 3.7. Explainability: Feature Contributions and Biological Plausibility

**Figure 6:**
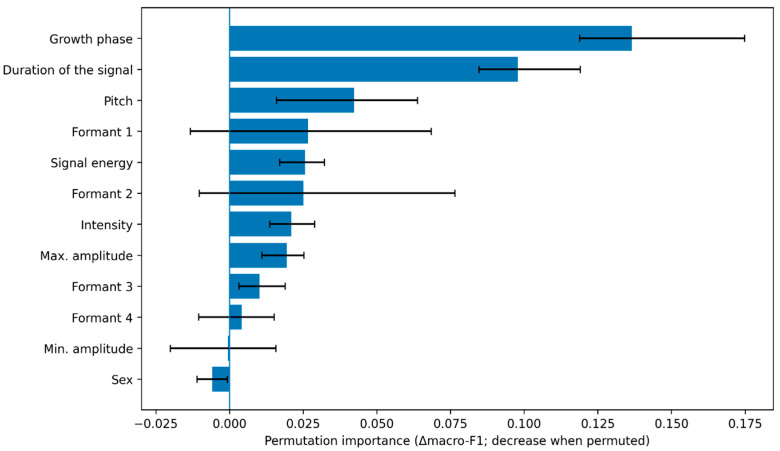
Permutation importance under Animal-grouped CV (top predictors).

Permutation importance under Animal-grouped cross-validation identified growth phase and temporal structure as the strongest contributors to generalization (Figure 6 and Table 5). The largest performance decrements were observed for the growth phase (mean Δmacro-F1 = 0.1365, 95% CI 0.1189–0.1748) and duration of the signal (Δmacro-F1 = 0.0978, 95% CI 0.0847–0.1191), followed by pitch (Δmacro-F1 = 0.0423, 95% CI 0.0160–0.0638). Energy- and amplitude-related descriptors (signal energy, intensity, and maximum amplitude) and spectral filtering measures (formants) provided additional, smaller but consistent contributions. In contrast, sex showed negligible importance (mean Δmacro-F1 < 0), consistent with the limited sex-related variance observed in the variance-partitioning analysis.

The importance profile suggests that developmental context (growth phase) and temporal organization (duration) are primary axes structuring the acoustic space. At the same time, distress separability is further supported by source-related features (energy/intensity/amplitude) and filter-related structure (formants). This pattern is biologically plausible: growth phase captures developmental changes in vocal production and vocal-tract morphology, whereas energy and intensity can reflect arousal-related vocal effort; formants likely reflect shifts in resonant filtering and source–filter coupling that vary with both development and affective state.

Across folds, intensity- and energy-related descriptors tended to rank highly, consistent with distress states that increase arousal and vocal effort. Spectral-resonance features (particularly higher formants) also showed reproducible contributions, supporting the interpretation that distress-related vocal production changes are not purely “loudness” effects but include changes in spectral filtering. In contrast, sex indicators showed comparatively small contributions, consistent with the limited ΔR^2^ attributed to sex in the variance-partitioning analysis. In contrast, the growth phase contributed indirectly by influencing baseline acoustic distributions and the confusability and structure observed across phases.

Because housing conditions and management practices are the dominant proximal causes of welfare deviations in commercial pig systems, we explicitly map each experimental exposure to plausible on-farm antecedents and actionable responses (Table 6). This framing emphasizes that acoustic classification is intended as decision support, an early warning that prioritizes targeted checks of ventilation, water delivery, feed access, and handling practices, rather than as a definitive diagnosis of a single, isolated stressor [1,2,3].

## 4. Discussion

Our findings support the central premise that stress-related vocal expression in pigs is not well represented by a single biomarker or a single categorical label but instead reflects a layered structure in which (a) within-animal individuality, (b) development/growth phase, and (c) distress context contribute separable, and partly interacting, components of variance. This aligns with the broader critique that stress is multidimensional and time-scale-dependent, and that “one-number” welfare assessments (including cortisol) often fail to differentiate stressor meaning or isolate causal drivers in ecologically realistic settings [2,3]. Empirically, our hierarchical decomposition provides a constructive alternative: rather than treating distress classes as error-free “truth,” it quantifies how much of each acoustic trait is plausibly attributable to distress exposure, developmental stage, and stable individual signatures.

### 4.1. Distress Effects in the Context of Multidimensional Stress and Measurement Limits

A key contribution of this study is that distress-related acoustic shifts were detectable while simultaneously accounting for individual identity and growth phase. This matters because physiological stress markers (e.g., cortisol) integrate multiple processes and can show heterogeneous or even weak correspondence with subjective or contextual stress reports, depending on timing and the pathway being indexed (e.g., acute reactivity vs. longer-term integration) [2]. More generally, stressor categories do not map uniquely onto cortisol responses, and cortisol dynamics may be similar across distinct stress modalities while differing in timing and context, limiting their interpretability as a stressor-specific classifier [17]. Against this background, the acoustic “fingerprints” you observed for distress exposures (including a comparatively clearer separation of pain from several non-pain challenges) reinforce the long-standing argument that vocalizations can serve as continuous, non-invasive welfare indicators when their structure–state links are properly modeled [18,19].

The acoustic shifts observed during heat stress, the elevation in pitch, and the reduction in call duration directly reflect the thermodynamic and physiological adjustments of the pigs. When environmental temperatures exceed the thermoneutral zone, pigs rely heavily on evaporative heat loss through panting. This increased respiratory rate (tachypnea) alters airflow dynamics through the vocal tract, constraining phonatory control and resulting in shorter, higher-pitched vocalizations. Likewise, the prominent acoustic overlap observed between hunger and heat stress highlights a critical threshold for acoustic-only monitoring systems. In commercial settings, this ambiguity can be resolved through a multimodal Precision Livestock Farming (PLF) approach. By fusing bioacoustic data with environmental sensors (temperature and humidity indices) and computer vision systems that detect stress-specific behaviors (such as spatial dispersion or lethargy during heat stress), systems can triangulate physiological demands, thereby validating welfare alerts and providing actionable, real-time decision support.

The overlap results (Figure 5, conceptually consistent with Figure 4) also carry an important theoretical implication: the strongest confusions are concentrated among physiologically adjacent non-pain challenges (notably Hunger–Heat stress and Normal–Thirst), rather than being uniformly distributed across all non-pain challenges. This pattern is consistent with a dimensional view of affect in which arousal and valence (and potentially motivational systems) overlap across contexts, leading to partially shared acoustic correlates even when the eliciting conditions differ [20,21]. The result model is not merely about “making mistakes”; it reveals the geometry of stress-like states as expressed in vocal features.

### 4.2. Developmental Modulation and Why the Growth Phase Must Be Explicitly Modeled

The observed phase-dependent trajectories (Figure 2) and the phase-stratified confusability structure indicate that distress encoding is developmentally reweighted. This resonates with two practical realities in pig production: animals experience multiple concurrent stressors throughout their lifespans, and the same stressor can manifest differently across developmental stages due to changes in physiology, body size, vocal-tract morphology, and behavioral repertoire [3]. Therefore, it is not surprising that later phases exhibited reduced classification margins and increased predictive entropy in our analysis. As animals mature, acoustic features linked to body size and call-production constraints can shift baseline distributions and reduce separability for certain distressors, particularly those that share arousal-related components [22].

From a methodological perspective, these results argue against a “phase-agnostic” approach to distress classification. Models trained across phases without a hierarchical structure risk conflating developmental variance with distress variance, leading either to inflated performance (if phase cues correlate with labels) or degraded generalization (if developmental distributions shift across farms or cohorts). The phase-specific confusions, therefore, provide a concrete justification for hierarchical pipelines in applied welfare monitoring: first, adapt to the animal’s developmental stage (or continuously model size/age), and only then classify distress subtypes within that stage.

### 4.3. Individual Signatures and the Scientific Meaning of ICC in Vocal Welfare Phenotyping

A second primary contribution is the magnitude and trait-specificity of individual identity effects, quantified via variance components and ICC. The fact that some traits exhibit non-trivial variance in “animal identity” is consistent with evidence that pig vocalizations encode stable aspects of emotional reactivity and individual differences across contexts [23]. This matters for both welfare science and deployment. Scientifically, it supports the concept that animals differ in baseline affective style/reactivity, such that two individuals may express similar distress contexts with different acoustic intensities or frequency profiles. In practice, this implies that precision welfare systems should not treat individuality as nuisance variance to ignore; rather, it can be used for personalized baselines, anomaly detection, and longitudinal welfare tracking.

The broader conceptual link to hierarchical modeling in the clinical/behavioral sciences is also clear: bifactor and hierarchical frameworks are explicitly designed to separate shared variance (a general factor) from domain-specific factors, thereby improving interpretability and construct validity when categories are heterogeneous and overlapping [9]. Translating that logic to animal emotion, the variance partitioning can be interpreted as an empirical decomposition into (1) general individual propensities (animal random effects), (2) developmental influences (phase), and (3) context-linked deviations (distress), which jointly generate the observed vocal phenotype.

### 4.4. Subject-Wise Generalization: The Reason Why a Grouped CV Is Essential

Our use of grouped cross-validation by animal is a critical methodological strength, as bioacoustic datasets are notoriously vulnerable to ‘identity leakage’ when samples from the same individual appear in both the training and test sets. Enforcing a strict subject-wise validation regime yields a realistic, conservative estimate of deployment utility on truly unseen animals. The resulting subject-wise performance, therefore, provides a more realistic estimate of deployment utility (i.e., performance on unseen animals). Within this strict generalization regime, the observed pattern, pain being relatively more separable while non-pain challenges show systematic overlap, should be framed as a scientifically informative result rather than merely a modeling limitation. In operational welfare terms, a monitoring system that reliably detects a pain-like distress signature and flags ambiguous non-pain states for secondary assessment could still have substantial value, especially when integrated with contextual sensors (temperature/humidity, water flow, feeding events) that disambiguate hunger/thirst/heat stress.

This point also connects to recent automation work in pig stress recognition that often reduces complex affective variation to binary “low/high stress” labels (e.g., facial-image DL classifiers), which can be useful but do not disentangle individual, developmental, and stressor-specific components [6,7]. Our results suggest a complementary approach: preserve the multi-class structure but explicitly represent the hierarchy (animal → phase → distress subtype) so that automation does not collapse biologically meaningful heterogeneity into a single axis.

### 4.5. Biological Plausibility and Alignment with Pig Vocalization Literature

Several aspects of our findings are consistent with prior studies on pig vocalization and reviews. First, the general feasibility of distinguishing distress contexts from vocal features is well established in pigs, including pain/cold/hunger discrimination in early life [18] and subsequent multi-stressor classification efforts incorporating heat and thirst [24]. Second, the broader farm-animal vocalization literature emphasizes that vocal output can provide continuous welfare-relevant information when call meaning and production constraints are respected [18]. Third, the field is increasingly consolidating around bioacoustics as a scalable component of precision livestock farming, while emphasizing methodological requirements for robust inference and generalization [25,26]. In this context, our contribution is not simply another classifier; it is an explanatory partition of variance that clarifies why some stressors are confusable (due to shared arousal-related correlates) and why developmental context reshapes separability.

### 4.6. Limitations and Recommended Next Steps

Because the dataset includes extracted features rather than raw audio, it does not allow testing self-supervised embeddings (e.g., wav2vec) or unsupervised call-type discovery (e.g., scream vs. grunt). Such approaches are a logical next step, as multi-level representations often outperform flat feature sets in emotion tasks when hierarchy is modeled explicitly [10]. Pitch missingness varied by growth phase and condition, so pitch effects likely reflect both genuine phonatory changes and increased unvoiced or low-quality segments in certain contexts. However, results were consistent across sensitivity analyses (complete-case vs. imputed; pitch included vs. excluded), and pitch-related findings, particularly in later phases and under hunger or heat stress, should be viewed as conservative.

Distress categories function as operational proxies; in reality, states may be continuous, overlapping, or context-dependent. This helps explain why categorical severity scoring can be subjective and difficult to standardize [4,5]. Accordingly, confusability patterns likely reflect partial state overlap rather than simple model error. The study involved 40 pigs under controlled conditions, so broader application requires external validation across farms, breeds, recording systems, and housing acoustics.

Future work should follow a hierarchy-aware roadmap: expand toward dimensional affect measures (e.g., arousal/valence) [21]; integrate contextual sensors (temperature, drinker, feeder data); apply hierarchical models that separate general distress from stressor-specific effects [9]; and validate on independent cohorts using animal-wise splits to preserve subject-level generalization.

### 4.7. Practical Implications for Welfare Monitoring and Decision Support

From an applied perspective, the results suggest a realistic, implementable strategy for precision welfare: use models as decision support rather than as single-shot labels. For example, a first-stage detector could identify “high likelihood pain-like distress” with high specificity. At the same time, a second stage, conditioned on growth phase and environment, could resolve hunger/thirst/heat stress with probabilistic outputs. This is consistent with the broader welfare-science argument that stress assessment should be multidimensional and context-aware, and that tools should not reduce complex states to simplistic severity bins [2,8].

While acoustic features offer a robust baseline for distress classification, the significant overlap between hunger and heat stress underscores the limitations of unimodal monitoring. The next step is to integrate acoustic data with complementary modalities, such as environmental sensors (e.g., temperature, humidity, feeding activity) and behavioral measures (e.g., movement or posture tracking). This multimodal approach would provide richer contextual information, improving discrimination between overlapping stressors and enhancing reliability in practical farm settings. By fusing continuous environmental indices (temperature and humidity) with computer-vision metrics (huddling or spatial dispersion), algorithms can cross-validate acoustic anomalies against environmental and behavioral data. This data fusion approach effectively disambiguates heat-induced tachypnea from resource-seeking vocalizations, ensuring high-accuracy, real-time decision support.

From an applied perspective, the present results indicate that acoustic monitoring can contribute to farm management by enabling earlier detection of welfare-relevant states, particularly those with clearer vocal signatures such as pain, and by supporting rapid investigation of conditions associated with thermal discomfort or resource restriction. In commercial systems, such early warning may assist decisions regarding ventilation adjustment, inspection of drinkers and feeders, regrouping, and handling practices, thereby improving animal welfare and helping to prevent secondary productivity losses. However, these benefits are indirect and depend on the management response triggered by the alert rather than on the acoustic signal itself.

In addition, acoustic thresholds should not be assumed to be universal across genetic groups. Because vocal production is influenced by body size, vocal-tract morphology, growth pattern, and stress responsiveness, breeds or lines may differ in baseline call structure and in the magnitude of acoustic changes under distress. Therefore, the present findings are most directly applicable to Landrace × Large White commercial pigs reared in intensive systems, and broader practical use will require external validation across breeds, farms, and housing conditions, as well as breed- and phase-specific calibration models.

For this reason, acoustic analysis should be viewed not as a stand-alone predictor of productivity but as one component of a precision livestock early-warning system whose main practical value lies in improving the timeliness and targeting of welfare interventions.

Future work should compare commercial and local breeds and establish breed- and phase-specific calibration models before broad on-farm deployment.

## 5. Conclusions

This study shows that pig vocalizations encode distress hierarchically, shaped by specific distress exposure, developmental stage, and stable individual signatures. Variance partitioning confirmed that, while distress and development are the primary drivers of acoustic variation, individual identity contributes a consistent, non-negligible influence, necessitating models that account for ‘voice identity’ to avoid performance inflation. From a practical perspective, this implies that bioacoustic indicators can be implemented either by focusing on universal markers of distress, such as alarm-type signals that are common across individuals, or by incorporating individual vocal identity to improve discrimination in overlapping stress conditions.

Under a rigorous subject-wise validation regime, which ensures generalization to unseen animals, the Random Forest classifier established a benchmark (macro-F1 = 0.597), significantly outperforming linear baselines and highlighting the importance of capturing non-linear feature interactions. While pain remains the most acoustically distinct condition, the structured overlap observed among non-pain stressors underscores the multidimensional nature of welfare. Ultimately, bioacoustics offers a scalable, non-invasive tool for precision livestock farming. However, the moderate performance observed underscores that effective deployment will depend on hierarchy-aware modeling and integration of contextual environmental data to improve reliability and resolve overlapping distress states.

## Figures and Tables

**Table 1:** Counts per growth phase × distress exposure × sex, with pitch missingness (*n*, %).

Growth Phase	Distress Exposure	Female	Male	Total	Missing Pitch, *n* (%)
Farrowing	Pain	60	60	120	0 (0.0)
	Hunger	61	60	121	0 (0.0)
	Cold	63	60	123	0 (0.0)
	Normal	59	59	118	0 (0.0)
Nursery	Pain	62	55	117	11 (9.4)
	Hunger	60	56	116	0 (0.0)
	Thirst	60	60	120	3 (2.5)
	Cold	60	63	123	0 (0.0)
	Normal	61	58	119	3 (2.5)
Growing	Pain	60	66	126	23 (18.3)
	Hunger	55	57	112	3 (2.7)
	Thirst	57	60	117	8 (6.8)
	Heat stress	57	58	115	16 (13.9)
	Normal	59	66	125	8 (6.4)
Finishing	Pain	56	56	112	18 (16.1)
	Hunger	49	56	105	66 (62.9)
	Thirst	59	55	114	0 (0.0)
	Heat stress	52	56	108	61 (56.5)
	Normal	54	56	110	21 (19.1)

**Table 2:** Descriptive statistics (mean ± SD) of key traits by distress exposure.

Distress Exposure	*n* (Total)	Intensity (dB)	Duration (s)	Pitch (Hz) Mean ± SD	*n* (Pitch)	Pitch Missing (%)	Formant 2 (Hz)
Pain	475	82.98 ± 7.09	0.94 ± 0.48	267.99 ± 172.49	423	10.9	2275.29 ± 252.02
Hunger	454	73.02 ± 6.64	0.64 ± 0.45	221.32 ± 124.33	385	15.2	1848.59 ± 237.92
Thirst	351	73.72 ± 5.40	0.52 ± 0.27	155.23 ± 135.65	340	3.1	1927.01 ± 287.97
Cold	246	74.36 ± 5.01	1.14 ± 0.78	151.42 ± 89.01	246	0.0	1768.13 ± 148.31
Heat stress	223	76.83 ± 5.69	0.34 ± 0.14	301.69 ± 102.99	146	34.5	1988.93 ± 206.36
Normal	472	72.76 ± 5.76	0.49 ± 0.29	171.53 ± 133.70	440	6.8	1900.99 ± 304.94

**Table 3:** Subject-wise (unseen-animal) model comparison and per-class performance for the best model. Overall performance (grouped 5-fold CV by Animal).

Model	Balanced Accuracy	Macro-F1
Random Forest	0.609	0.597
Gradient Boosting	0.566	0.565
Multinomial logistic regression	0.552	0.498

**Table 4:** Random Forest class-wise precision/recall/F1 (grouped-CV pooled predictions).

Class	Precision	Recall	F1	Support
Normal	0.563	0.464	0.509	472
Pain	0.832	0.825	0.829	475
Hunger	0.569	0.509	0.537	454
Thirst	0.499	0.556	0.526	351
Cold	0.634	0.663	0.648	246
Heat stress	0.463	0.637	0.536	223

**Table 5:** Permutation importance under Animal-grouped cross-validation (top predictors).

Rank	Feature	Mean Δmacro-F1	Title 1	CI2.5	CI97.5
1	Growth phase	0.1365	entry 1	0.1189	0.1748
2	Duration of the signal	0.0978		0.0847	0.1191
3	Pitch	0.0423		0.0160	0.0638
4	Formant 1	0.0267		−0.0133	0.0685
5	Signal energy	0.0257		0.0171	0.0323
6	Formant 2	0.0251		−0.0103	0.0766
7	Intensity	0.0210		0.0136	0.0289
8	Max. amplitude	0.0194		0.0110	0.0253
9	Formant 3	0.0102		0.0033	0.0189
10	Formant 4	0.0041		−0.0105	0.0152
11	Min. amplitude	−0.0006		−0.0201	0.0158
12	Sex	−0.0059	entry 2	−0.0110	−0.0007

Obs. Permutation importance is reported as the mean decrease in macro-F1 (Δmacro-F1) when each predictor is permuted in the held-out animals. Uncertainty is summarized as the 2.5^th^ and 97.5^th^ percentiles across grouped folds.

**Table 6:** Mapping of experimental distress exposures to plausible on-farm antecedents and recommended management responses in commercial housing systems.

Experimental Condition (Study Label)	Typical on-Farm Antecedent (Housing/Management)	Rapid Checks (What to Verify First)	Immediate Management Response (Mitigation)	Preventive (Housing/Management)
Heat stress	Ventilation failure; inadequate air exchange; cooling system malfunction; high stocking density; heat waves; high humidity limiting evaporative cooling.	Fans/inlets/exhaust functioning; temperature–humidity; air speed at animal level; wet floors/litter; crowding; water availability.	Restore ventilation and airflow; activate cooling (pads/misting where appropriate); reduce handling; provide additional water access; redistribute animals to reduce crowding.	Preventive maintenance of fans/inlets; heat-load protocols; alarm thresholds; stocking density management; contingency plans for power/equipment failure [1,3].
Cold stress	Drafts; inadequate insulation; heating failure; wet flooring/bedding; insufficient microclimate control (especially young pigs).	Draft detection; floor temperature; moisture/wetness; heater function; pig posture/huddling.	Reduce drafts; provide dry bedding; restore heating; improve microclimate; adjust ventilation to balance air quality and warmth.	Building envelope improvements; zone heating; dry-floor management; seasonal ventilation settings [1].
Thirst (water restriction)	Drinker blockage/malfunction; insufficient flow rate; poor drinker placement; competition at drinkers; water supply interruption.	Flow rate per drinker; blockage/leaks; number of drinkers per pig; water pressure; water line function; evidence of queuing.	Repair/flush drinkers; increase drinker availability; adjust placement; ensure uninterrupted supply; consider regrouping if competition is severe.	Water system monitoring and maintenance; redundant drinker points; routine flow audits; design to reduce competition [1,2].
Hunger (feed restriction/competition)	Feeder malfunction; rationing errors; insufficient feeder space; diet transition problems; dominance-related competition; delayed feeding.	Feeder function and access; feed availability; feeder space per pig; feed delivery schedule; queueing/aggression at feeder.	Restore feed access; adjust feed delivery; add feeder space or multiple feeding points; reduce competition (e.g., regrouping or pen design changes).	Feeder design and stocking protocols; feed delivery QC; management to reduce competition and mixing stress [1,3].
Pain/noxious handling stimulus	Rough handling; improper restraint; injury/lameness; aggressive interactions; iatrogenic pain during routine procedures (where relevant).	Handling procedures; injury check; lameness scoring; body lesion assessment; review recent interventions.	Stop/modify handling; treat injured animals; isolate severe cases; review procedure protocols; refine staff training.	Staff training and handling standardization; injury prevention; protocol refinement for welfare-critical procedures; severity assessment frameworks and oversight [3,4,5].
Normal (baseline)	Standard husbandry and stable microclimate with adequate resources.	Verify environment within target ranges; confirm feed/water access; observe behavior.	Maintain routine; use as baseline for comparison and drift detection.	Continuous monitoring to detect deviation from baseline; documentation of housing/management changes to contextualize alerts [2].

## Data Availability

The original contributions presented in this study are included in the article/Supplementary Material. Further inquiries can be directed to the corresponding author(s).

## Supplementary Materials

The following supporting information can be downloaded at: https://www.mdpi.com/article/10.3390/ani16081148/s1, Table S1: Marginal intraclass correlation coefficient (ICC) for animal identity. File S1: The dataset used in this study.
