# Supplementary material for: Hierarchical Acoustic Encoding Distress in Pigs: Disentangling Individual, Developmental, and Emotional Effects with Subject-Wise Validation
Source: Animals (Basel). 2026 Apr 9;16(8):1148. doi: 10.3390/ani16081148 (PMC13113075; doi:10.3390/ani16081148)
Supplement: Supplementary file 1 [file animals-16-01148-s001.zip › Table S1.pdf]

Supplementary Table S1. Marginal intraclass correlation coefficient (ICC) for animal identity**.**

Trait	Unit	N	Var_fixed	Var_animal (from residuals)	Var_residual (from residuals)	ICC_conditional	ICC_marginal
Intensity	dB	2221	22.7254	1.1076	29.1633	0.0366	0.0209
Duration	s	2221	0.089	0.002	0.1605	0.0124	0.008
Pitch	Hz	1980	4083.0486	123.2152	16635.8034	0.0074	0.0059
Formant 2	Hz	2221	40303.8329	1661.7841	51485.714	0.0313	0.0178

Note: Marginal ICC was computed as Var(Animal)/(Var(Fixed)+Var(Animal)+Var(Residual)), where Var(Fixed) is the variance of fitted values from the fixed-effects model (Sex + Growth phase + Distress exposure), and Var(Animal) and Var(Residual) were estimated from residual variance components across animals. Conditional ICC is shown for reference as Var(Animal)/(Var(Animal)+Var(Residual)).
